# Nutritional Quality of Plant-Based Cheese Available in Spanish Supermarkets: How Do They Compare to Dairy Cheese?

**DOI:** 10.3390/nu13093291

**Published:** 2021-09-21

**Authors:** Ujué Fresán, Holly Rippin

**Affiliations:** 1 eHealth Group, Instituto de Salud Global Barcelona (ISGlobal), 08036 Barcelona, Spain; 2 Independent Nutritionist, Cumbria LA22, UK; rippinholly@gmail.com

**Keywords:** dairy alternative, dairy substitute, cheese analogues, vegan cheese, vegetarian cheese, plant-based alternatives

## Abstract

Plant-based cheese is one of the most increasingly consumed dairy alternatives. Evidence is lacking on their nutritional quality. We aimed to evaluate the nutritional composition of the plant-based cheese options available in Spanish supermarkets, and how they compare with dairy cheese. An audit of plant-based cheese alternatives has been conducted in seven of the most common supermarkets. For each product, the nutritional content per 100 g and ingredients were collected. Data on generic dairy cheese were retrieved from the BEDCA website. Descriptive statistics (median, minimum and maximum) were used to characterize the plant-based cheese products, for both all the products and grouped by main ingredients (i.e., coconut oil, cashew nuts and tofu). Mann–Whitney U tests were used for comparisons between dairy and different types of plant-based cheese. The coconut oil-based products (the large majority of plant-based cheese products, *n* = 34) could not be considered as healthy foods. Their major ingredients were refined coconut oil and starches and were high in saturated fats and salt. The other smaller groups, cashew nut- (*n* = 4) and tofu-based (*n* = 2), showed a healthier nutritional profile. Replacing dairy cheese with these groups could be nutritionally beneficial. Future investigations should address the health effects of substituting dairy cheese with these products.

## 1. Introduction

An increasing number of people in developed countries, including in Europe, are moving away from animal-rich diets to adopt plant-based dietary patterns [1]. Different reasons are behind this transition, such as animal welfare, environmental degradation and health, among others [2,3,4]. The general adoption of plant-based diets has been highlighted as one of the most effective options for mitigating climate change [5], and a necessary shift if we are to feed the 10 billion people expected by 2050 within planetary boundaries [6]. At the same time, the general adoption of plant-based diets could benefit the health of populations globally [7,8]. While the consumption of animal-sourced foods such as red and processed meat has been linked to detrimental health outcomes such as colorectal cancer, cardiovascular disease and diabetes [9], the health benefits of whole plant-based foods, such as legumes, whole grains, nuts, vegetables and fruits, have also been reported in the scientific literature [9]. Indeed, replacing animal products with whole plant-based options has been linked to health benefits [10,11].

With the aim of facilitating this dietary transition, more and more plant-based products imitating animal-sourced equivalents are reaching the market, such as meat and dairy analogs [12,13]. Moreover, forecasts suggest that their consumption will continue growing [12,13]. This is especially relevant in Europe; four out of five of the top countries in the world with the highest share of global vegan new product launches in food and drink in 2018 were European, namely Germany, the United Kingdom, France and Spain [1]. The emergence and increased popularity of these products is concerning public health and nutrition professionals. Although some studies have aimed to assess how nutritionally sound and healthy these options are, they are relatively few, and the World Health Organization (WHO) has called for more studies on the topic [14]. The investigations carried out to date have been focused on the assessment of plant-based meat and milk alternatives [15,16,17,18], while less attention has been paid to other food products, such as plant-based cheese.

A recent Spanish report assessing those eating a plant-based diet found that 43% of interviewees had eaten plant-based cheese during the last three months. Indeed, plant-based cheese is one of the most consumed plant-based alternatives, after milk, meat and yogurts [19]. Evidence is lacking on the nutritional adequacy of plant-based cheese alternatives. The aim of this study is, therefore, to evaluate the nutritional and ingredient composition of plant-based cheese options available in Spanish supermarkets and compare the nutrient composition with that of dairy cheese.

## 2. Materials and Methods

### 2.1. Plant-Based Cheese Alternatives

An audit of plant-based cheese alternatives was conducted in seven of the most common supermarkets in Spain, namely Carrefour, Mercadona, Alcampo, Día, Lidl, Eroski and El Corte Inglés, to reflect choices available to the majority of Spanish consumers. The websites of those supermarkets were assessed during April and May 2021, using the keywords “queso vegano” and “queso vegetal” (“vegan cheese” and “plant-based cheese”, in Spanish, respectively) to ensure all available products were captured. Supermarkets were visited in person to capture any additional products that were sold in situ but not online. For each product, several data were collected: name, commercial brand, supermarkets in which the product was available, selling format (i.e., block, slices, grated, spreads), nutritional content and ingredients. Nutritional data (i.e., calories, total fat, saturated fat, carbohydrates, sugars, fiber, protein and salt per 100 g of product) and ingredient lists were obtained from the nutritional label on the commercial package or from the information located on the website of the retailer. Retail data for all products were double checked against data from the website of the producer of each product. Supplementary Table S1 shows the main ingredient, selling format, calories and nutritional content per 100 g of the assessed plant-based cheese alternatives.

### 2.2. Dairy Cheese Data

Data on the most commonly consumed generic types of dairy cheese in Spain were retrieved from the BEDCA (Spanish food composition database) website [20]. The format in which each cheese types are regularly sold in stores is not specifically reported for all products. In cases where this was missing, the lead author assigned the most common format according to their experience. One of the following three formats was selected: block/slices, grated and spreads. The BEDCA does not report nutrition information about sugars content in foods. Dairy cheese products formatted as block/slices have a content of total carbohydrates of 0 g/100 g of cheese; only two products are reported as having 0.3 and 0.5 g of carbohydrates per 100 g. We, therefore, assigned a sugars value of 0 g for all block/slices products. However, the two examples of dairy spreadable cheese had 2.3 and 3.1 g of carbohydrates per 100 g of cheese. The researcher in charge of the database confirmed that all carbohydrates in those spreadable products were sugars (personal communication); thus, the sugars content in those products was considered as 2.3 and 3.1 g per 100 g, respectively. As salt content is reported as mg of sodium per 100 g, sodium values were multiplied by 2.5 and divided by 1000 in order to obtain g of salt per 100 g of product. Specific types of dairy cheese were considered; their selling format (i.e., block/slices, grated, spreads) and their caloric and nutritional values are reported in Supplementary Table S2.

### 2.3. Statistical Analysis

Normality of data distribution was tested through the Shapiro–Wilk test and rejected. Descriptive statistics (median and range) of energy and selected nutrients (total fats, saturated fats, carbohydrates, sugars, fiber, protein and salt) per 100 g were stated to describe plant-based cheese products. This characterization was performed for all plant-based cheese products as a whole, and also by groups. Groups were assigned according to their main ingredients (i.e., coconut oil, cashew nuts and tofu) and within each of these groups, by selling format (i.e., block/slices, grated and spreads). Comparisons between dairy and plant-based cheese were carried out using the Mann–Whitney U test for independent samples. We compared all dairy products to all plant-based alternatives as a whole, and also by group (by main ingredient and selling format). The statistical analysis was performed through the statistical software jamovi (version 1.6) [21], with the significance level set at *p* < 0.05.

## 3. Results

### 3.1. Plant-Based Cheese Alternatives

In total, 40 different plant-based cheese products were detected. One supermarket had by far the largest product offer, selling 38 out of the 40 assessed products; the other supermarkets had a much lower number of products available (*n* = 5, 3, 1, 1, 0 and 0). The most common selling formats were blocks (*n* = 15), slices (*n* = 10), grated (*n* = 8) and spreads (*n* = 7). The products fell into three different categories regarding their ingredients’ composition: the largest group (82.5%, *n* = 34) was mainly composed of refined coconut oil and starches; two smaller groups had cashew nuts (*n* = 4) and tofu (*n* = 2) as the major ingredients. Not all the coconut oil-based products reported on the percentage of coconut oil they contain. In those reporting that value, the coconut oil content ranged between 20–29%. In the case of cashew nut-based products, around 50% of their weight were cashews, followed by water and lemon juice. In the case of tofu-based products, 98.5% of the product weight was soy milk. The coconut oil-based products contained many food additives including thickeners, preservatives, flavorings and colorings. In the case of the cashew nut- and tofu-based products, there were fewer additives, primarily natural flavorings, and agar-agar (a gelling agent) in cashew nut-based options. Six commercial brands manufactured coconut oil-based products, while all the cashew nut-based and tofu-based products were produced by a single company each. Supplementary Table S1 shows the main ingredient, selling format, energy and nutritional content per 100 g of the assessed plant-based cheese alternatives.

Overall, the plant-based cheese alternatives did not have a good nutritional profile (Table 1) [22,23,24]. They were high in calories (median: 288 kcal/100 g), fats (median: 23 g/100 g), saturated fats (median: 20 g/100 g) and salt (median: 1.5 g/100 g), while they contained a low amount of protein (median: 0.5/100 g) and fiber (median: 0 g/100 g). Assessing the products grouped by major ingredient, the coconut oil-based cheese (Table 1) contained a median of 287 kcal/100 g. They were high in fats (median: 23 g/100 g), being mainly saturated fats (median: 21 g/100 g) and salt (median: 1.6 g/100 g). Their median carbohydrate content was 20 g/100 g, with negligible sugars. They contained a very low amount of protein (median: 0.4 g/100 g) and no fiber. Assessing the nutritional composition of the coconut oil-based products by selling format (i.e., blocks/slices, grated and spreads), the same general pattern was observed in the three groups, except for the spreadable products, which had a lower amount of carbohydrates (Supplementary Table S3). The cashew nut-based cheese products (Table 1) were energy-dense, with a median of 323 kcal/100 g. They were high in fats (median: 25 g/100 g), and their saturated fat content was 5.7 g/100 g. They were a good source of protein, with 11 g/100 g, 2.7 g/100 g of (natural) sugars, and 2.6 g/100 g of fiber. Their salt content was moderate (0.6 g/100 g). All the cashew nut-based products were sold as blocks; thus, no analysis by selling format was carried out. The tofu-based cheese products (Table 1) provided a median of 185 kcal, 11 g of total fats and 1.7 g of saturated fats per 100 g of product. They were a good source of protein (median: 18 g/100 g) and fiber (median: 6.2 g/100 g). Their median amount of salt was 1.0 g/100 g. As in the case of the cashew nut-based options, all the tofu-based cheese were sold as blocks; therefore, no analysis by selling format was carried out.

### 3.2. Plant-Based Cheese Alternatives vs. Dairy Cheese

The dairy cheese products were higher in calories (median: 364 vs. 288 kcal (*p* < 0.001)), total fats (median: 31 vs. 23 g (*p* < 0.001)) and proteins (median: 23 vs. 0.5 g (*p* < 0.001)), per 100 g, than the plant-based cheese alternatives. Dairy cheese was lower in carbohydrates (median: 0 vs. 19.9 g (*p* < 0.001)), sugars (*p* = 0.002) and fiber (*p* < 0.001). There were no significant differences in the amount of saturated fats and salt between the dairy cheese and the plant-based alternatives (*p* > 0.05) (Table 2).

When assessing the differences by type of products according to the major ingredient, the nutritional composition of the coconut oil-based products was quite similar to that of dairy products, aside from being significantly lower in protein (*p* < 0.05). The products sold as blocks or slices were slightly lower in calories (367 vs. 289 kcal/100 g (*p* < 0.001)) and total fats (31.3 vs. 23 g/100 g (*p* < 0.001)), but not in saturated fatty acids (*p* = 0.392) (Table 3). As shown in Table 4, compared to the dairy cheese, both the cashew nut-based and tofu-based products were the less caloric options (cashew nut-based: 367 vs. 328 kcal/100 g (*p* = 0.007); tofu-based: 367 vs. 185 kcal/100 g (*p* = 0.024)) and lower in fats, both total (cashew nut-based: 31.3 vs. 25 g/100 g (*p* = 0.009); tofu-based: 31.3 vs. 11 g/100 g (*p* = 0.024)) and especially saturated fats (cashew nut-based: 19.4 vs. 5.7 g/100 g (*p* = 0.002); tofu-based: 19.4 vs. 1.7 g/100 g (*p* = 0.023)). Their salt content was also lower (cashew nut-based: 1.7 vs. 0.6 g/100 g (*p* = 0.002); tofu-based: 1.7 vs. 1.0 g/100 g (*p* = 0.045)). As expected, their fiber content was higher (*p* < 0.05). The cashew nut-based products had more sugars (0 vs. 2.7 g/100 g (*p* < 0.001)), and less protein (23.4 vs. 11.0 g/100 g (*p* = 0.002)) than dairy cheese. No difference was observed among the amount of protein between the tofu-based and milk-based cheese options (*p* = 0.143).

## 4. Discussion

The present study describes the ingredients and nutritional profile of the plant-based cheese alternatives currently available in Spanish supermarkets and how they compare nutritionally to dairy cheese. Our findings indicate that the availability of plant-based cheese products is relatively low; only one supermarket had a considerable number of options (*n* = 38). However, the rest of the supermarkets have at the disposal of customers few products, if any. This is in accordance with the fact that Spanish plant-based dieters demand for a greater offer of these products [19]. Our findings also support their concern for the low nutritional quality of available products [19]. The majority of these products were high in calories, fats, especially saturated fat and salt, while they were low in fiber and proteins. Therefore, these products would not be considered healthy plant-based products [22,23,24]. However, not all the products had the same ingredients’ composition or nutritional profile.

Coconut oil-based cheese (the largest group of available products) had a no healthy nutritional profile. Those plant-based products were mainly composed of refined coconut oil with some starches. They were, therefore, high in saturated fats. No significant difference was observed between the saturated fat content of dairy cheese and the coconut oil-based alternatives. The evidence suggests that limiting the consumption of foods high in saturated fats is necessary for health reasons [25]. Specifically, the cardiovascular health benefits of consuming virgin coconut oil, which is high in saturated fats, have been disproved [26,27]. In addition, the coconut-oil based products were made of refined fats, of which consumption should be limited [28]. However, coconut oil is preferred by the plant-based cheese industry because its high amount of saturated fatty acids enables a creamy texture and also gives firmness to the product at refrigerated temperatures. Additional ingredients such as starches, carrageenan or agar-agar are also used in coconut oil-based cheese to mimic the density and texture of real cheese [29,30]. Refined coconut oil is preferred by the industry over its virgin counterpart because it is less intense in flavor and could be easily masked by the use of flavorings [30,31]. This may explain the presence of several food additives in those products. The coconut oil-based products were also high in salt, with over 1.5 g salt per 100 g [22,23]. High salt intake has been recognized as one of the main diet-related risk factors for global mortality and for loss of quality-adjusted life years [32]. These alternatives also contained negligible amounts of protein; therefore, they cannot be considered as a dietary protein source, as dairy cheese is.

The ingredient composition of cashew nut-based and tofu-based products seem nutritionally healthier. In the case of cashew nut-based products, around 50% of their weight were cashews, being the major ingredient, followed by water and lemon juice. The health benefits of whole nuts consumption is well-known [33,34], and it has been reported that other nut-based processed products, such as peanut butter without added sugars, could provide some health benefits, reducing the risk of type 2 diabetes incidence [35]. In the case of tofu-based products, 98.5% of the products’ weight is soy milk. Similarly, some studies suggest that tofu has health benefits for type 2 diabetes prevention [36] and soy milk consumption for dyslipidemia management [37]. Further studies could investigate whether cashew nut-based and tofu-based cheese can also improve people’s health.

The difference in the calorie content between dairy cheese and cashew nut-based options, besides being statistically different, is minimal. However, the replacement of dairy cheese by tofu-based alternatives could help reduce energy intake. In addition, the replacement of dairy cheese with cashew nut-based and particularly tofu-based options may be helpful in reducing the intake of total fats, and would contribute to replacing the intake of saturated fats by unsaturated options, which could provide health benefits [25]. However, unsaturated fatty acids are sensitive to processing, being easily oxidized. Lipid oxidation lowers the nutritional quality of lipid-containing foods [38,39]. Future analysis should determine to what extent the lipid profile of the primary ingredients of these food products is affected by processing. The cashew nut-based and tofu-based products were good sources of protein (median: 11 g and 18 g/100 g for cashew nut-based and tofu-based, respectively) [24]. Indeed, there is no significant difference in the protein level of dairy cheese and tofu-based alternatives. In addition, soy protein could also be considered as a complete protein, containing all the essential amino acids. On the other hand, the substitution of dairy cheese with cashew nut-based options would moderate the intake of protein. Considering that a large proportion of Spaniards have an excessive intake of proteins [40], along with the fact that the distribution of macronutrients in diets of Spanish vegetarians and vegans corresponds well to that proposed by the Spanish standards for dietary reference intake, including the protein content [41], the replacement of dairy cheese by cashew nut-based alternatives would not necessarily be a problem in terms of dietary protein intake if integrated in a nutritionally balanced diet. They could also provide some fiber, especially tofu-based products. Fiber consumption has been linked to several health benefits, such as a reduced risk of all-cause and cardiovascular-related mortality, and incidence of coronary heart disease, stroke incidence and mortality, type 2 diabetes and colorectal cancer, among others [42]. Both types of products contained lower levels of salt compared to dairy cheese, although the salt content in tofu-based cheese was relatively high [23].

Altogether, the cashew nut-based and tofu-based products currently available in Spanish supermarkets seem to provide an overall nutritionally healthier profile and could be interesting substitutes for dairy cheese. However, they are not a common option; only four cashew nut-based and two tofu-based cheese products were available in supermarkets at the time of the study, and just one out of the seven stores visited sold them. Our findings, along with the fact that Spanish people eating plant-based diets demand more and healthier plant-based cheese products in supermarkets [19], indicate that there is room for the food industry to commercialize new plant-based cheese options with a better nutritional profile. Currently, just one brand manufactures each of those healthier cashew and tofu-based options for Spanish supermarkets. New nut- and tofu-based products to be marketed should avoid the addition of unhealthy ingredients, such as refined oils, starches and excess salt that would offset the benefits of using those healthy main ingredients. However, the food industry may face some barriers in reformulating foods, such as higher costs or lower consumer acceptability if the organoleptic properties do not sufficiently mimic dairy cheese. More efforts into mitigating this is needed [43,44].

It should be noted, however, that not all plant-based cheese consumers opt for these alternatives for health reasons, but for other reasons such as environmental or animal welfare motives. The evidence suggests that plant-based meat and milk analogs have a lower environmental impact than the animal-based products they are intended to replace [17,45,46]. To the authors’ knowledge, the ecological footprint of plant-based cheese has not been addressed in the scientific literature. A Danish database reports that the carbon footprint of producing 1 kg of vegan cheese would be 1 kg of carbon dioxide equivalents (CO_2_ e) [47], a value significantly lower than that assigned to dairy cheese, ranging from 5.33 to 16.35 kg CO_2_ e per kg of product [48]. Without concrete data, it can only be assumed that—as long as the main ingredients (i.e., coconut oil, cashew and soy) are grown using low environmental impact agricultural techniques—these plant-based products would be environmentally friendlier alternatives to dairy cheese, which is one of the food products with the highest impact on the environment [46].

A major strength of our study is that, for the first time, the nutritional profile and ingredients’ composition of the plant-based cheese alternatives available in Spanish supermarkets has been carried out. In addition, their nutritional composition has been compared with that of dairy cheese. Our results debunk the common assumption that all plant-based products are healthy options, highlighting the necessity of assessing other plant-based products on the market. Some limitations should be also considered. Only products available at supermarkets were targeted, not considering those products exclusively sold in specialist vegan stores; this study’s objective was to assess the products available for consumers on a mass scale. This would be a good area for future research. Further research could also investigate to what extent the protein and lipid profile of ingredients have been affected by processing [38,39,49]. Other data gaps, such as the micronutrient content, particularly those that are characteristic of dairy cheese, such as calcium, also deserve further attention.

## 5. Conclusions

This study shows that the majority of plant-based cheese alternatives available in Spanish supermarkets do not have a good nutritional profile. Nevertheless, although relatively few products are available, healthier options could be found, such as those goods composed mainly of cashew nuts and tofu. The replacement of dairy cheese by cashew nut- and tofu-based plant-based alternatives could reduce intakes of salt and total fats, while replacing the intake of saturated with unsaturated fats. Future investigations should address the health effects of substituting dairy cheese with plant-based cheese products. The assessment of the environmental impact of plant-based cheese also deserves further attention.

## Figures and Tables

**Table 1:** Median (minimum-maximum) values of calories and nutritional content in plant-based cheese alternatives per 100 g, by ingredients’ composition.

	All Products *n* = 40	Coconut Oil-Based *n* = 34	Cashew Nut-Based *n* = 4	Tofu-Based *n* = 2
	Median (Min–Max)	Median (Min–Max)	Median (Min–Max)	Median (Min–Max)
Calories (kcal)	288 (185–328)	287 (200–327)	328 (306–328)	185 (185–185)
Total fat (g)	23.0 (11.0–29.0)	23.0 (16.7–29.0)	25.0 (21.0–25.9)	11.0 (11.0–11.0)
Saturated fat (g)	20.0 (1.7–26.0)	21.0 (8.3–26.0)	5.7 (4.4–6.3)	1.7 (1.7–1.7)
Carbohydrate (g)	19.9 (0.5–30.0)	20.0 (1.3–30.0)	13.3 (11.9–17.1)	0.5 (0.5–0.5)
Sugars (g)	0.2 (0.0–7.10)	0.1 (0.0–7.1)	2.7 (2.7–3.5)	0.0 (0.0–0.0)
Fibre (g)	0.0 (0.0–6.2)	0.0 (0.0–5.9)	2.6 (2.2–2.7)	6.2 (6.2–6.2)
Protein (g)	0.5 (0.0–18.0)	0.4 (0.0–6.0)	11.0 (10.6–11.0)	18.0 (18.0–18.0)
Salt (g)	1.5 (0.5–3.5)	1.6 (1.0–3.5)	0.6 (0.5–0.6)	1.0 (1.0–1.0)

**Table 2:** Median (minimum–maximum) values of calories and nutritional content in dairy cheese and plant-based cheese alternatives per 100 g.

	Dairy Cheese *n* = 22	Plant-Based Cheese *n* = 40	*p* Value
	Median (Min–Max)	Median (Min–Max)	
Calories (kcal)	364 (201–467)	288 (185–328)	<0.001
Total fat (g)	31 (11.2–40.50)	23.0 (11.0–29.0)	<0.001
Saturated fat (g)	18.9 (7.0–25.4)	20.0 (1.7–26.0)	0.729
Carbohydrate (g)	0.0 (0.0–3.1)	19.9 (0.5–30.0)	<0.001
Sugars (g)	0.0 (0.0–3.1)	0.2 (0.0–7.10)	0.002
Fibre (g)	0.0 (0.0–0.0)	0.0 (0.0–6.2)	<0.001
Protein (g)	23.0 (6.5–32.30)	0.5 (0.0–18.0)	<0.001
Salt (g)	1.7 (0.1–3.8)	1.5 (0.5–3.5)	0.492

Mann–Whitney U non-parametric test for independent samples was used to perform comparisons among dairy and plant-based cheese. *p* < 0.05 is considered statistically significant.

**Table 3:** Median (minimum-maximum) values of calories and nutritional content in dairy cheese and coconut oil-based cheese alternatives per 100 g, by format.

	Block/Slices			Grated			Spreads		
	Dairy Cheese *n* = 17	Coconut Oil-Based *n* = 19	*p* Value	Dairy Cheese *n* = 3	Coconut Oil-Based *n* = 8	*p* Value	Dairy Cheese *n* = 2	Coconut Oil-Based *n* = 7	*p* Value
	Median (Min–Max)	Median (Min–Max)		Median (Min–Max)	Median (Min–Max)		Median (Min–Max)	Median (Min–Max)	
Calories (kcal)	367 (201–467)	290 (273–327)	<0.001	367 (223–395)	289 (240–313)	0.473	306 (251–361)	267 (200–290)	0.500
Total fat (g)	31.3 (11.2–40.5)	23.0 (17.0–29.0)	<0.001	26.5 (16.1–32.1)	21.8 (16.7–25.9)	0.473	28.2 (23.9–32.4)	25.0 (16.7–28.8)	0.500
Saturated fat (g)	19.4 (7.0–25.4)	20.9 (16.0–26.0)	0.392	16.7 (8.7–17.1)	19.5 (8.3–22.0)	0.357	17.0 (14.3–19.6)	23.0 (15.0–24.2)	0.184
Carbohydrate (g)	0.0 (0.0–0.5)	20.0 (6.1–30.0)	<0.001	0.0 (0.0–0.0)	21.4 (18.3–26.7)	0.017	2.7 (2.3–3.1)	6.0 (1.3–13.30)	0.186
Sugars (g)	0.0 (0.0–0.0)	0.1 (0.0–7.1)	<0.001	0.0 (0.0–0.0)	0.0 (0.0–0.2)	0.292	2.7 (2.3–3.1)	0.2 (0.0–1.1)	0.053
Fibre (g)	0.0 (0.0–0.0)	0.0 (0.0–5.9)	0.014	0.0 (0.0–0.0)	0.0 (0.0–4.0)	0.449	0.0 (0.0–0.0)	0.0 (0.0–2.2)	0.384
Protein (g)	23.4 (13.8–29.0)	0.5 (0.0–2.2)	<0.001	26.9 (19.5–32.3)	0.0 (0.0–1.6)	0.014	11.1 (6.5–15.6)	0.2 (0.0–6.0)	0.053
Salt (g)	1.7 (0.7–3.8)	1.8 (1.0–3.5)	0.514	1.5 (0.9–2.1)	1.9 (1.0–2.3)	0.474	1.2 (0.1–2.3)	1.2 (1.0–1.6)	1.000

Mann–Whitney U non-parametric test for independent samples was used to perform comparisons among dairy and plant-based cheese. *p* < 0.05 is considered statistically significant.

**Table 4:** Median (minimum-maximum) values of calories and nutritional content of those dairy cheese and cashew nut- and tofu-based cheese alternatives formatted as block and slices per 100 g.

	Dairy Cheese *n* = 17	Cashew Nut-Based *n* = 4	*p* Value	Dairy Cheese *n* = 17	Tofu-Based *n* = 2	*p* Value
	Median (Min–Max)	Median (Min–Max)		Median (Min–Max)	Median (Min–Max)	
Calories (kcal)	367 (201–467)	328 (306–328)	0.008	367 (201–467)	185 (185–185)	0.028
Total fat (g)	31.3 (11.2–40.5)	25 (21–25.9)	0.011	31.3 (11.2–40.5)	11.0 (11.0–11.0)	0.028
Saturated fat (g)	19.4 (7.0–25.4)	5.7 (4.4–6.3)	0.003	19.4 (7.0–25.4)	1.7 (1.7–1.7)	0.028
Carbohydrate (g)	0.0 (0.0–0.5)	13.3 (11.9–17.1)	<0.001	0.0 (0.0–0.5)	0.5 (0.5–0.5)	0.008
Sugars (g)	0.0 (0.0–0.0)	2.7 (2.7–3.5)	<0.001	0.0 (0.0–0.0)	0.0 (0.0–0.0)	NaN
Fibre (g)	0.0 (0.0–0.0)	2.5 (2.2–2.7)	<0.001	0.0 (0.0–0.0)	6.2 (6.2–6.2)	<0.001
Protein (g)	23.4 (13.8–29.0)	11.0 (10.6–11.0)	0.003	23.4 (13.8–29.0)	18.0 (18.0–18.0)	0.163
Salt (g)	1.7 (0.7–3.8)	0.6 (0.5–0.6)	0.003	1.7 (0.7–3.8)	1.0 (1.0–1.0)	0.053

NaN: not a number. Mann–Whitney U non-parametric test for independent samples was used to perform comparisons among dairy and plant-based cheese. *p* < 0.05 is considered statistically significant.

## Data Availability

Data is contained within the article or supplementary material.

## Supplementary Materials

The following are available online at https://www.mdpi.com/article/10.3390/nu13093291/s1, Table S1: Main ingredient, format, calories and nutritional content per 100 g of plant-based cheese alternatives, Table S2: Format, calories and nutritional content per 100 g of different types of dairy cheese, Table S3: Median (minimum-maximum) values of calories and nutritional content in coconut oil-based cheese alternatives per 100 g, by selling format.
