# Supplementary material for: Nutritional Quality of Plant-Based Cheese Available in Spanish Supermarkets: How Do They Compare to Dairy Cheese?
Source: Nutrients. 2021 Sep 21;13(9):3291. doi: 10.3390/nu13093291 (PMC8465168; doi:10.3390/nu13093291)
Supplement: Supplementary file 1 [file nutrients-13-03291-s001.zip › nutrients-1325670-supplementary.pdf]

Supplementary Table S1. Main ingredient, format, calories and nutritional content per 100 g of plant-based cheese alternatives

Product id	Main ingredient	Format	Calories (kcal)	Total fats (g)	Saturated fats (g)	Carbohydrates (g)	Sugars (g)	Fibre (g)	Protein (g)	Salt (g)
1	Coconut oil	Grated	298	24.0	22.0	21.0	0.0	0.0	0.0	2.2
2	Coconut oil	Block	305	29.0	26.0	11.0	0.0	0.0	0.0	1.7
3	Coconut oil	Grated	304	22.9	19.0	21.7	0.2	4.0	0.7	1.8
4	Coconut oil	Block	303	21.5	17.6	26.4	3.1	0.5	1.0	1.5
5	Coconut oil	Block	297	20.8	17.4	23.7	0.0	5.9	0.5	1.8
6	Coconut oil	Block	303	24.4	20.9	19.5	0.1	1.5	0.5	1.6
7	Coconut oil	Slices	288	21.9	21.2	20.7	0.2	0.0	2.2	1.0
8	Coconut oil	Slices	288	21.9	21.2	20.7	0.2	0.0	2.2	1.0
9	Coconut oil	Slices	293	23.0	20.0	20.0	0.0	0.0	1.4	2.0
10	Coconut oil	Slices	285	23.0	21.0	20.0	0.0	0.0	0.0	2.3
11	Coconut oil	Slices	285	23.0	21.0	20.0	0.0	0.0	0.0	2.3
12	Coconut oil	Slices	290	23.0	21.0	21.0	1.2	0.0	0.5	2.3
13	Coconut oil	Block	276	25.3	16.0	6.1	0.4	5.4	0.0	1.5
14	Coconut oil	Block	327	26.8	21.5	18.6	0.5	4.7	0.4	1.5
15	Coconut oil	Grated	267	16.7	8.3	26.7	0.0	0.0	0.0	2.0
16	Coconut oil	Grated	267	16.7	10.0	26.7	0.0	0.0	0.0	1.9
17	Coconut oil	Grated	313	25.9	21.6	18.3	0.0	3.2	0.0	1.6
18	Coconut oil	Grated	280	20.6	19.9	22.6	0.2	0.0	1.6	1.0
19	Coconut oil	Grated	240	17.0	16.6	20.0	0.2	0.0	1.6	1.0
20	Coconut oil	Slices	273	22.7	18.2	18.2	0.0	0.0	0.0	1.9
21	Coconut oil	Spreads	267	23.3	15.0	13.3	0.0	0.0	0.0	1.0
22	Coconut oil	Spreads	200	16.7	15.0	13.3	0.0	0.0	0.0	1.6
23	Coconut oil	Spreads	290	28.8	24.2	3.9	0.6	1.3	3.4	1.3
24	Coconut oil	Spreads	283	27.6	23.4	5.2	1.0	1.8	2.5	1.0
25	Coconut oil	Spreads	286	28.0	23.0	1.3	1.1	2.2	6.0	1.2
26	Coconut oil	Spreads	246	25.0	23.0	6.0	0.2	0.0	0.2	1.5
27	Coconut oil	Spreads	239	23.0	21.0	8.0	0.0	0.0	0.0	1.2
28	Coconut oil	Block	311	23.7	20.0	22.7	7.1	2.1	0.6	1.5

29	Coconut oil	Block	303	24.0	22.0	20.0	0.0	0.0	0.0	2.2
30	Coconut oil	Block	279	17.0	16.0	30.0	0.3	0.0	1.6	3.5
31	Coconut oil	Slices	297	24.0	20.7	19.8	0.0	0.0	0.3	1.5
32	Coconut oil	Slices	285	23.0	21.0	20.0	0.0	0.0	0.0	2.3
33	Coconut oil	Grated	298	24.0	22.0	21.0	0.0	0.0	0.0	2.3
34	Coconut oil	Slices	284	22.0	20.0	20.0	1.0	0.0	1.2	2.2
35	Cashew nuts	Block	306	21.0	4.4	17.1	3.5	2.4	11.0	0.5
36	Cashew nuts	Block	328	25.0	6.3	13.3	2.7	2.7	11.0	0.6
37	Cashew nuts	Block	328	25.0	6.3	13.3	2.7	2.7	11.0	0.6
38	Cashew nuts	Block	328	25.9	5.1	11.9	2.7	2.2	10.6	0.5
39	Tofu	Block	185	11.0	1.7	0.5	0.0	6.2	18.0	1.0
40	Tofu	Block	185	11.0	1.7	0.5	0.0	6.2	18.0	1.0

Supplementary Table S2. Format, calories and nutritional content per 100 g of different types of dairy cheese

Type of cheese	Format	Calories (kcal)	Total fats (g)	Saturated fats (g)	Carbohydrates (g)	Sugars (g)	Fibre (g)	Protein (g)	Salt (g)
Blue cheese	Block/slices	351	29.8	17.6	0.0	0.0	0.0	21.1	2.0
Brie cheese	Block/slices	343	30.7	19.4	0.0	0.0	0.0	17.2	1.5
Goat cheese, cured	Block/slices	464	39.6	25.4	0.0	0.0	0.0	27.6	2.0
Cabrales cheese	Block/slices	377	32.6	19.4	0.0	0.0	0.0	21.5	2.7
Camembert cheese, 20-30% fidm[a]	Block/slices	201	11.2	7.0	0.0	0.0	0.0	24.7	2.2
Camembert cheese, 60% fidm[a]	Block/slices	350	33.1	20.7	0.0	0.0	0.0	13.8	1.3
Cheddar cheese	Block/slices	405	33.6	21.3	0.1	0.0	0.0	26.0	1.8
Ball cheese	Block/slices	336	28.3	17.3	0.0	0.0	0.0	20.7	1.9
Lamb, cow and goat cheese, "Castilla-la Mancha" type	Block/slices	413	35.7	23.3	0.0	0.0	0.0	23.5	1.1
Sheep and cow cheese, "Castilla-León" type	Block/slices	410	35.8	23.3	0.0	0.0	0.0	22.6	1.7
Cheese, edam type	Block/slices	336	28.3	17.3	0.0	0.0	0.0	20.7	1.1
Emmental cheese	Block/slices	367	28.8	17.3	0.0	0.0	0.0	27.3	0.7
Gruyere cheese	Block/slices	375	31.3	18.8	0.4	0.0	0.0	23.4	1.1
Cheese, "manchego" type, in oil	Block/slices	467	40.5	24.5	0.0	0.0	0.0	26.5	1.8
Roquefort cheese	Block/slices	354	32.8	20.6	0.0	0.0	0.0	15.4	3.8
Semi-cured cheese	Block/slices	390	30.4	18.9	0.5	0.0	0.0	29.0	1.7
Fresh cheese	Block/slices	332	25.4	13.7	0.0	0.0	0.0	26.0	1.7
Mozzarella cheese	Grated	223	16.1	8.7	0.0	0.0	0.0	19.5	0.9
Parmesan cheese	Grated	367	26.5	16.7	0.0	0.0	0.0	32.3	2.1
Grated cheese	Grated	395	32.1	17.1	0.0	0.0	0.0	26.9	1.5
Cheese spread	Spreads	361	32.4	19.6	2.3	2.3	0.0	15.6	2.3
Cheese spread, with spice and herbs	Spreads	251	23.9	14.3	3.1	3.1	0.0	6.5	0.1

[a]fidm: fat in dry matter

Supplementary Table S3. Median (minimum-maximum) values of calories and nutritional content in coconut oil-based cheese alternatives per 100g, by selling format

	**Block/slices**<br>n=19	**Grated**<br>n=8	**Spreads**<br>n=7
	Median (Min-Max)	Median (Min-Max)	Median (Min-Max)
Calories (kcal)	290 (273-327)	289 (240-313)	267 (200-290)
Total fat (g)	23.0 (17.0-29.0)	21.8 (16.7-25.9)	25.0 (16.7-28.8)
Saturated fat (g)	20.9 (16.0-26.0)	19.4 (8.3-22.0)	23.0 (15.0-24.2)
Carbohydrate (g)	20.0 (6.1-30.0)	21.4 (18.3-26.7)	6.00 (1.3-13.3)
Sugars (g)	0.1 (0.0-7.1)	0.0 (0.0-0.2)	0.2 (0.0-1.1)
Fibre (g)	0.0 (0.0-5.9)	0.0 (0.0-4.0)	0.0 (0.0-1.1)
Protein (g)	0.5 (0.0-2.2)	0.0 (0.0-1.6)	0.2 (0.0-6.0)
Salt (g)	1.8 (1.0-3.5)	1.9 (1.0-2.3)	1.2 (1.0-1.6)
